# Supplementary material for: Metabolism of the Cyanogenic Glucosides in Developing Flax: Metabolic Analysis, and Expression Pattern of Genes
Source: Metabolites. 2020 Jul 14;10(7):288. doi: 10.3390/metabo10070288 (PMC7407305; doi:10.3390/metabo10070288)
Supplement: Supplementary file 1 [file metabolites-10-00288-s001.zip › Table S2.doc]

**Table S2.** The activity of GC methabolism genes during plant growth in control and Met25 expressing plants.

.

| **CONTROL PLANT-LINOLA** | | | | | | | | |
| --- | --- | --- | --- | --- | --- | --- | --- | --- |
| DAS | **Val oxy** | | **Linamarase** | | **HNL** | | **CAS** | |
|  | mean | SE | mean | SE | mean | SE | mean | SE |
| 5 | **0.54** | 0.02 | **0.69** | 0.01 | **0.09** | 0.01 | **0.69** | 0.03 |
| 20 | **0.77** | 0.03 | **0.93** | 0.06 | **1.20** | 0.08 | **1.33** | 0.02 |
| 30 | **0.35** | 0.01 | **0.80** | 0.05 | **0.76** | 0.05 | **0.87** | 0.08 |
| 55 | **1.81** | 0.09 | **0.98** | 0.091 | **0.13** | 0.01 | **0.60** | 0.01 |
| 65 | **2.01** | 0.10 | **5.00** | 0.24 | **3.02** | 0.75 | **1.71** | 0.22 |
| 85 | **0.19** | 0.02 | **11.85** | 0.889 | **1.03** | 0.09 | **2.15** | 0.78 |
| **Met25** | | | | | | | |  |
| DAS | **Val oxy** | | **Linamarase** | | **HNL** | | **CAS** | |
|  | mean | SE | mean | SE | mean | SE | mean | SE |
| 5 | **0.25** | 0.01 | **0.69** | 0.01 | **1.02** | 0.04 | **1.31** | 0.02 |
| 20 | **0.74** | 0.03 | **0.76** | 0.03 | **0.98** | 0.02 | **1.66** | 0.04 |
| 30 | **0.53** | 0.03 | **0.90** | 0.03 | **0.56** | 0.02 | **1.24** | 0.02 |
| 55 | **1.31** | 0.91 | **1.14** | 0.91 | **0.30** | 0.01 | **1.75** | 0.33 |
| 65 | **1.37** | 0.09 | **3.46** | 0.13 | **2.33** | 0.34 | **5.41** | 0.56 |
| 85 | **0.21** | 0.01 | **9.21** | 0.51 | **0.46** | 0.01 | **6.15** | 0.74 |

The mRNA level of GC methabolism genes (Val-monooxygenase, Linamarase, β-cyanoalanine synthase (CAS) and hydroxynitrilase (HNL)) Real-time (RT-PCR) analysis. Data represent the mean ± standard deviations from three independent experiments
