# Supplementary material for: Metabolism of the Cyanogenic Glucosides in Developing Flax: Metabolic Analysis, and Expression Pattern of Genes
Source: Metabolites. 2020 Jul 14;10(7):288. doi: 10.3390/metabo10070288 (PMC7407305; doi:10.3390/metabo10070288)
Supplement: Supplementary file 1 [file metabolites-10-00288-s001.zip › Table S1.doc]

**Table S1.** Content of cyanogenic glycosides (mg/gDW) in flax tissues at subsequent stages of development of control plants (Linola) and those producing sulfur amino acids (Met25).

| **CONTROL PLANT-LINOLA** | | | | | | | | |
| --- | --- | --- | --- | --- | --- | --- | --- | --- |
| DAS | **Linustatin** | | **Linamarin** | | **Neolinastatin** | | **Lotaustralin** | |
|  | mean | SE | mean | SE | mean | SE | mean | SE |
| 0 | **4.096** | 0.122 | **1.152** | 0.036 | **6.495** | 0.402 | **0.624** | 0.009 |
| 3 | **1.401** | 0.048 | **5.618** | 0.298 | **2.950** | 0.259 | **2.114** | 0.264 |
| 7 | **0.427** | 0.011 | **26.979** | 0.945 | **0.314** | 0.008 | **20.368** | 0.012 |
| 14 | **0.301** | 0.006 | **24.654** | 0.762 | **0.214** | 0.017 | **18.237** | 0.014 |
| 21 | **0.107** | 0.002 | **17.324** | 0.551 | **0.114** | 0.001 | **9.076** | 0.081 |
| 35 | **0.115** | 0.002 | **7.623** | 0.299 | **0.141** | 0.001 | **6.680** | 0.149 |
| 49 | **0.086** | 0.001 | **8.289** | 0.612 | **0.139** | 0.002 | **7.046** | 0.205 |
| 56 | **0.067** | 0.001 | **18.161** | 0.992 | **0.094** | 0.001 | **9.328** | 0.099 |
| 70 | **0.061** | 0.002 | **18.344** | 0.946 | **0.088** | 0.001 | **7.086** | 0.303 |
| 100 | **0.065** | 0.001 | **1.530** | 0.046 | **0.027** | 0.001 | **0.999** | 0.021 |
| 120 | **2.985** | 0.020 | **1.060** | 0.024 | **4.239** | 0.200 | **0.626** | 0.025 |
| **Met25 PLANT** | | | | | | | | |
| DAS | **Linustatin** | | **Linamarin** | | **Neolinastatin** | | **Lotaustralin** | |
|  | mean | SE | mean | SE | mean | SE | mean | SE |
| 0 | **4.045** | 0.43 | **1.247** | 0.039 | **7.022** | 0.406 | **0.646** | 0.006 |
| 3 | **2.538** | 0.052 | **2.635** | 0.303 | **4.571** | 0.459 | **0.982** | 0.168 |
| 7 | **0.846** | 0.062 | **27.130** | 0.933 | **1.235** | 0.022 | **13.936** | 0.387 |
| 14 | **0.431** | 0.008 | **25.610** | 0.986 | **0.673** | 0.016 | **11.981** | 0.017 |
| 21 | **0.127** | 0.001 | **17.143** | 0.545 | **0.138** | 0.001 | **9.198** | 0.118 |
| 35 | **0.135** | 0.002 | **8.922** | 0.249 | **0.090** | 0.001 | **6.776** | 0.155 |
| 49 | **0.112** | 0.001 | **9.789** | 0.923 | **0.130** | 0.001 | **6.693** | 0.198 |
| 56 | **0.099** | 0.001 | **13.654** | 0.799 | **0.170** | 0.001 | **6.321** | 0.152 |
| 77 | **0.084** | 0.002 | **17.670** | 0.834 | **0.137** | 0.001 | **5.709** | 0.303 |
| 100 | **0.045** | 0.001 | **0.999** | 0.069 | **0.017** | 0.001 | **0.988** | 0.002 |
| 120 | **2.899** | 0.100 | **1.027** | 0.025 | **4.899** | 0.333 | **0.700** | 0.028 |

Data represent the mean (± standard deviations) from at least three independent experiments. The significance of the differences between the means was determined using Student’s test (P < 0.05)
