# Supplementary material for: Metabolism of the Cyanogenic Glucosides in Developing Flax: Metabolic Analysis, and Expression Pattern of Genes
Source: Metabolites. 2020 Jul 14;10(7):288. doi: 10.3390/metabo10070288 (PMC7407305; doi:10.3390/metabo10070288)
Supplement: Supplementary file 1 [file metabolites-10-00288-s001.zip › Table S3.doc]

**Table S3. Sequences of primers used in RT-PCR analysis**

| **Target gene** | **Primer forward** | | **Primer reverse** |
| --- | --- | --- | --- |
| **Reference gene** | | | |
| Actin | 5' CCGGTGTTATGGTTGGAAT 3' | | 5' TGTAGAAAGTGTGATGCCAAA 3' |
| **CG metabolism genes** | | | |
| Val- monooxygenase | 5' CAGGTAGCATTGTGACTGGC 3' | 5' CCAGCACCGGTATGATGTTG 3' | |
| Linamarase | 5' TCCCAGAACGACATTGAGG 3' | 5' TGAACGGAAAGGCAACGGTA 3' | |
| hydroxynitrilase (HNL) | 5' GGAAAGCCAAGACCGTGATTA 3' | 5' GACCTCGTACTTGCACTTCATAG 3' | |
| β-cyanoalanine synthase (CAS) | 5' CAAATTACCGGCAATGGAGT 3' | 5' AGCAAGTTCAAGTGCTGCAA 3' | |
