# Supplementary material for: Metabolism of the Cyanogenic Glucosides in Developing Flax: Metabolic Analysis, and Expression Pattern of Genes
Source: Metabolites. 2020 Jul 14;10(7):288. doi: 10.3390/metabo10070288 (PMC7407305; doi:10.3390/metabo10070288)
Supplement: Supplementary file 1 [file metabolites-10-00288-s001.zip › Supp Fig1.doc]

**Supp. Fig 1.** Schematic representation of changes in cyanogenic monoglucoside level and flavonoids (apigenin and luteolin) content during development of flax (control) plant.(uper panel) and the mRNA level of valine monoxygenase (NADPH oxidoreductase) and flavonoid 3′-hydroxylase (F3′H).

DAS – days after sowing
